# Recent Advances in Costimulatory Blockade to Induce Immune Tolerance in Liver Transplantation

**DOI:** 10.3389/fimmu.2021.537079

**Published:** 2021-02-24

**Authors:** Mingjie Ding, Yuting He, Shuijun Zhang, Wenzhi Guo

**Affiliations:** ^1^ Department of Hepatobiliary and Pancreatic Surgery, The First Affiliated Hospital of Zhengzhou University, Zhengzhou, China; ^2^ Open and Key Laboratory of Hepatobiliary & Pancreatic Surgery and Digestive Organ Transplantation at Henan Universities, Zhengzhou, China; ^3^ Zhengzhou Key Laboratory of Hepatobiliary & Pancreatic Diseases and Organ Transplantation Medicine, Zhengzhou, China

**Keywords:** liver transplantation, immune tolerance, costimulatory, block, induce

## Abstract

Liver transplantation is an effective therapy for end-stage liver disease. However, most postoperative patients must take immunosuppressive drugs to prevent organ rejection. Interestingly, some transplant recipients have normal liver function and do not experience organ rejection after the withdrawal of immunosuppressive agents. This phenomenon, called immune tolerance, is the ultimate goal in clinical transplantation. Costimulatory molecules play important roles in T cell-mediated immune responses and the maintenance of T cell tolerance. Blocking costimulatory pathways can alter T cell responses and prolong graft survival. Better understanding of the roles of costimulatory molecules has facilitated the use of costimulatory blockade to effectively induce immune tolerance in animal transplantation models. In this article, we review the state of the art in costimulatory pathway blockade for the induction of immune tolerance in transplantation and its potential application prospects for liver transplantation.

## Introduction

Liver transplantation is the most effective treatment for end-stage liver disease. However, graft rejection seriously restricts graft function and recipient quality of life. The emergence of immunosuppressive agents has reduced the occurrence of rejection and improved transplant outcomes. However, most recipients require lifelong immunosuppression, which is expensive and increases the risk of infection; additionally, for hepatocellular carcinoma patients, immunosuppressants increase the risk of tumor recurrence after transplantation. As an “immune-privileged organ,” the liver has a lower probability and degree of rejection after transplantation than many solid organs (1). Indeed, in the clinical setting, some transplant recipients develop liver graft immune tolerance-normal liver graft function in the absence of graft rejection after the withdrawal of immunosuppression. The induction of immune tolerance is the ultimate goal for transplant doctors, as it is the best way to avoid graft rejection and the toxic side effects caused by immunosuppressive agents.

Earlier studies found that approximately 20% of liver transplant patients developed immune tolerance after they stopped taking immunosuppressants (2) among those who could not successfully stop taking the drugs, some patients were able to take lower doses of their immunosuppressants. In 2012, Feng et al. (3) conducted an immunosuppressive drug withdrawal test on 20 pediatric patients who had received related-living-donor liver transplants. They found that up to 60% of the recipients successfully stopped taking immunosuppressants completely and achieved liver transplantation immune tolerance. Moreover, the later the time of drug withdrawal after surgery, the higher the probability of the recipient achieving immune tolerance. In an international multicenter study of 102 adult liver transplant patients, 41.8% of those followed for more than 5 years successfully stopped taking immunosuppressants (4). In another study of adult liver transplant patients, up to 63% successfully stopped taking immunosuppressants and achieved immune tolerance (5). Liver transplantation recipients typically achieve immune tolerance late after transplantation, whereas they mainly experience adverse reactions to immunosuppressive agents in the early period after transplantation. Therefore, it is critical to intervene early after transplant to help recipients develop immune tolerance and avoid the deleterious effects of immunosuppressive drugs. The mechanism of liver transplantation immune tolerance has not been fully elucidated, so most methods for inducing immune tolerance are still in preclinical or clinical stages of experimentation.

## The Roles and Mechanisms of T Cells in Immune Tolerance

Immune tolerance is divided into central and peripheral immune tolerance. Central immune tolerance is the tolerance to autoantigens generated by exposure to those antigens during embryonic development and the development of T and B cells. Peripheral immune tolerance occurs when mature T cells and B cells are exposed to endogenous or exogenous antigens in the absence of the signals that lead to an immune response. The liver has excellent immune regulation abilities that ensure local and systemic immune tolerance to self and foreign antigens, as well as the effective immune response to pathogens, and immune tolerance is a dynamic, self-replicating state, which requires the host to recognize the graft antigen to form a stable regulatory environment (6). Liver transplant rejection is the core content of transplantation immunity research, and it is an adaptive immune response that involves the activation of T and B lymphocytes. T cells play an important role in immune responses to allografts, the activation of T cells can lead to rejection of allografts, but sometimes it will weaken in the process of liver transplantation, which can promote the acceptance of transplanted liver and even immune tolerance. The mechanism of induction and maintenance of tolerance has been the main focus of transplant immunology researchers.

After the body recognition of “non-self” antigens, immune cells can be activated and generate appropriate immune responses through a series of cell responses, including proliferation and differentiation (7). However, the immune cells showed low or no response when faced self-antigens, and this non-responsive situation or state can be considered as immune tolerance. The formation and maintenance of immune tolerance are affected by multiple immune cells, and T cells act as the most important role, which are the major player of the adaptive immune system. T cells can be divided into different subgroups according to their function, mainly including CD4+T cells (helper T cells, Th), CD8+T cells (cytotoxic T cells, Tc), suppressor T cells, etc. CD4 + cells have affinity for MHC class II, while CD8 + cells have affinity for MHC class I. Th cells can be divided into Th1 and Th2 subsets, in normal conditions, Th1/Th2 is in dynamic balance. Th1 cells mainly secrete interleukin-2 (IL-2), interferon-γ (IFN-γ), tumor necrosis factor-β (TNF-β) and other cytokines, they can activate Tc to induce delayed type hypersensitivity, and can also activate macrophages and natural killer (NK) cells to specifically kill the antigen of the grafts, and participate in the cellular immune response. Th2 cells mainly secrete cytokines such as IL-4, IL-5, IL-10 and IL-13, which participate in humoral immune responses. Meanwhile, they can also induce specific cellular immune responses through other pathways. Th1 and Th2 cells restrict each other. IL-10 can inhibit the synthesis of Th1 cytokines, especially IFN-γ, while IFN-γ can selectively inhibit the proliferation of Th2 cells. Th1 cells play an important role in the development of acute rejection after liver transplantation, while Th2 is mainly related to the formation of tolerance, and the deviation fromTh1 to Th2 is considered to be one of the mechanisms of transplantation tolerance.

Tolerance can be defined as the graft receptor cannot express the destructive immune response of the graft, which can be described as a complex process, balancing the reactivity against foreign antigens and autoantigens. T cell tolerance is an unresponsive state of T cells to self-antigens to prevent the occurrence of autoimmune diseases. Under the stimulation of T cell receptor (TCR) signal, the tolerant T cells could not effectively proliferate and secrete cytokines. There are two different mechanisms for the T cells tolerance occurs. The first is the exhaustion of self-reactive T cells during their maturation in the thymus and the other is to inhibit and/or elimination of self-reactive mature T cells in the periphery (8). T cells need to undergo negative selection and positive selection during their maturation in the thymus gland, and eventually become mature CD4+ and CD8+T cells. After negative and positive selection, mature T cells (CD4+and CD8+) are released from the thymus into the peripheral circulation and secondary lymphoid organs. Most self-reactive T cells are eliminated in the thymus by negative selection, however, it is incomplete and a certain number of self-reactive T cells that escape negative selection and migrate to the periphery. These escaped self-reactive T cells can be eliminated in the periphery through a series of tolerance mechanisms, including the induction of anergy (unresponsiveness), suppression by other immunologically active cells (Tregs) and deletion. T cells activation or tolerance is regulated by a series of costimulatory signals, on one hand, such as CD28 and inducible costimulator (ICOS) are important costimulatory molecules required for T cells activation and function, and inhibit or deficiencies in both them can lead to T cells tolerance. On the other hand, many inhibitory costimulatory molecules such as CTLA-4, PD-1, Lag-3, Tigit, B7-H3,BTLA and B7S1 can also regulate T cells activation or tolerance (9, 10). When T cells are stimulated by TCR and receive a large amount of inhibitory costimulatory signals and lack of positive costimulatory signals, it will lead to T cell tolerance, which is mainly manifested as limited cell expansion and impaired effector function (11, 12).

## T-Cell Activation and Costimulatory Molecular Pathways

**Figure 1:**
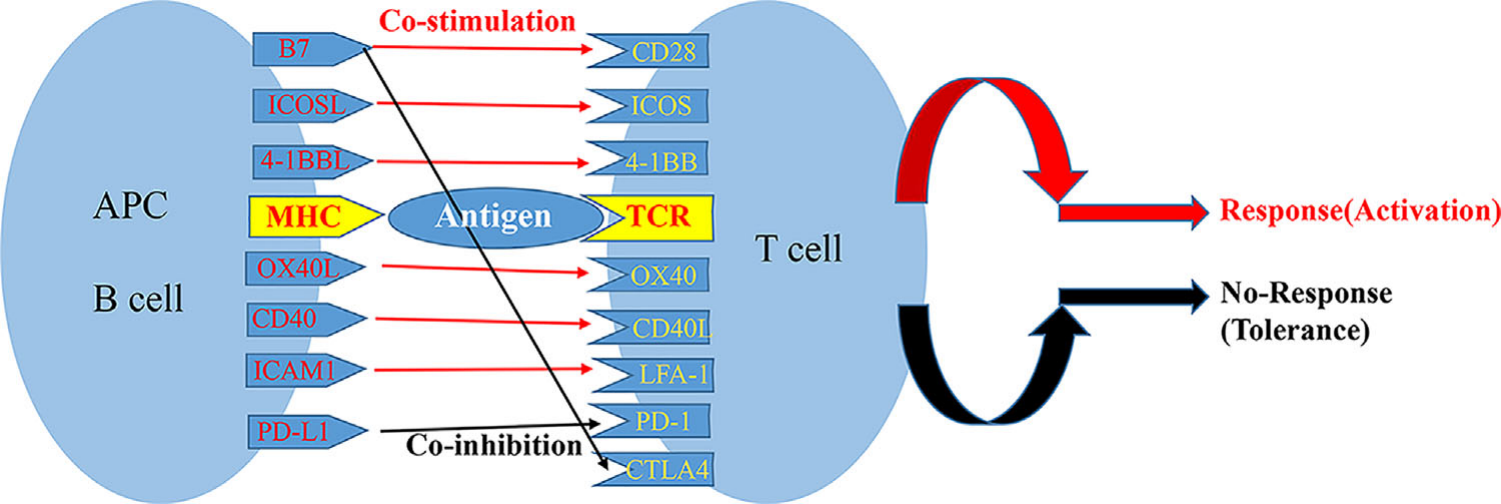
Major costimulatory molecular pathways and their interplay.

The activation of T cells is a complex process, which three signals are typically required to fully activate T cells. The first signal is specific binding of the TCR on the surface of the initial T cell to an antigen peptide: major histocompatibility complex on the surface of an antigen-presenting cell (APC) (13). The second signal is the interaction of a costimulatory receptor on the T cell membrane with its ligand on the surface of the APC; these costimulatory pairs include CD28/B7 ligands (B7-1 and B7-2), CD40/CD40 ligand (also known as CD154), tumor necrosis factor (TNF) receptor superfamily member 4 (also known as OX40)/TNF superfamily member 4 (also known as OX40L), and ICOS/ICOS ligand (ICOSLG). The balance of signals from costimulatory and coinhibitory receptors on the surface of a T cell determines the functional result of TCR signal transduction (14). TCR stimulation in the absence of the second signal can result in anergy, immune tolerance, or even programmed cell death (**Figure 1**). When the costimulatory signal exceeds the coinhibitory signal, transcription factors are activated that trigger the production of IL-2 and other proinflammatory factors, thereby promoting T cell proliferation and differentiation.

Based on their structures, costimulatory molecules can be roughly divided into 4 groups: the immunoglobulin (Ig)-related family, the TNF-related family, the hepatitis A virus cellular receptor 2 (also known as T-cell immunoglobulin mucin family member 3 [TIM]) family, and the adhesion factor family. In general, the Ig-related superfamily and TNF-related super families are particularly important for adaptive immune responses (15). These costimulatory molecular pathways play important roles in the recognition of antigens and the activation of T cells.

The inhibition of costimulatory molecules is essential for the establishment and maintenance of peripheral immune tolerance. In the absence of appropriate costimulation, the recognition of an antigen by a TCR makes the T cell non-responsive to the antigen, thereby inducing peripheral tolerance (16). Multiple mechanisms contribute to the formation of transplant tolerance, including ignorance, deletion, anergy, exhaustion, and immune regulation; nearly all of these mechanisms involve alloreactive T cells. As blocking these second signals can prevent T cell activation and acute rejection, costimulatory blockade is currently one of the most active areas of research in transplantation immunity. Studies have shown that blocking the activation of T cells can prolong graft survival time (17). So, blocking costimulatory pathways during liver transplantation may change anti-allograft immune responses and weaken rejection, and it is may be a strategy to induce immune tolerance in transplant recipients, thereby limiting toxicity from immunosuppressive drugs after transplantation (18). So, we review the current state of costimulatory pathway blockade for the induction of immune tolerance in transplantation (summarized in **Table 1**).

**Table 1:** The roles of costimulatory pathways in liver transplantation.

Costimulatory Signal	Ligand	Strategies to target	Outcome (Effects on liver transplantation immune tolerance)	References
CD28	B7	CTLA-4Ig	Suppress T cell dependent immune response and prolong the long-term survival of xenografts and allografts.	(19–22)
		(belatacept)	Animal Trials: Successfully induced immune tolerance.	(23)
			Clinical Trials: Phase II showed acute rejection and graft loss.	(24)
ICOS	ICOSL	anti- ICOS mAb	Prolong the survival of rat liver allografts and prevent acute rejection, and the combination with FK506 can induce grafts tolerance.	(25) (26)
		RNAi- ICOS	Prevents acute rejection and prolongs the survival of grafts.	(27)
CD40	CD154	anti-CD40 mAb (ASKP1240)	Animal Trials: The non-human primates showed good tolerance and increased the survival rate of liver grafts.	(28)
			The anti-CD40 mAb can prolong the survival of xenografts.	(29)
OX40	OX40L	OX40Ig	Inhibit the rejection of allografts and induce immune tolerance by reducing IL-2 expression.	(30)
4-1BB	4-1BBL	anti-4-1BB mAb	Prolong the allograft survival time and prevent allograft rejection.	(31)
		RNAi-4-1BB	Inhibiting or alleviating acute rejection of liver transplantation in rats.	(32, 33)
GITR	GITRL		Still to be explored in liver transplantation.	
Tim-1		anti-Tim-1 mAb	3B3: Promote T cell proliferation and block allograft tolerance.	(34, 35)
		(3B3, MT1-10)	MT1-10: No application has been found in liver transplantation.	
Tim-3	galectin-9	anti-Tim-3 mAb (RMT3-23)	No sufficient data.	
Tim-4		anti-Tim-4 mAb	Alleviate the acute rejection injury and down-regulate the expression of pro-inflammatory factors.	(36)
LFA-1	ICAM-1	anti-LFA-1 mAb anti-ICAM-1 mAb	Prolong the allografts survival time, but can not induce permanent tolerance.	(37)

## Ig-Related Superfamily Costimulatory Pathways

### CD28/B7 Costimulatory Pathway

CD28/B7 is the most important and best-studied costimulatory pathway in transplantation. CD28, the most important costimulatory molecule in the T cell membrane, is a homodimeric cell surface glycoprotein that belongs to the Ig transmembrane superfamily (38). CD80 (also known as B7-1) and CD86 (also known as B7-2), the ligands of CD28, are also members of the Ig superfamily. B7-1 exists as a dimer on the cell surface, whereas B7-2 is a monomer. CD28 binding to B7-1 and B7-2 on APCs activates CD28 signal transduction to enhance T cell responses to antigens. This signal promotes T cell proliferation through the transcription of cytokines such as IL-2 and enhances T cell survival through the transcription of Bcl2-Bclx (39). After the activation of T cells, they can express cytotoxic T-lymphocyte associated antigen 4 (CTLA4), which also binds B7-1 and B7-2. Unlike CD28, CTLA4 is a negative regulatory factor that sends inhibitory signals to T cells, thereby limiting the T cell responses. CTLA4 shares sequence similarity with CD28, for which it is a structural analog. CTLA4 competitively binds to B7-1/B7-2 with higher affinity than CD28, thereby blocking costimulatory signals.

CTLA4Ig (belatacept) is a soluble fusion protein that was approved by the Food and Drug Administration in 2011 for use in renal transplantation patients. It blocks the CD28/B7 pathway in T cells, inhibits T cell activation, and promotes graft tolerance. *In vivo* experiments have shown that CTLA4Ig suppresses T cell-dependent immune responses and prolongs the long-term survival of xenografts and allografts (19–21). CTLA4Ig can markedly prolong the survival of allografts in non-human primates (NHPs) (22). Two phase III clinical trials found that the overall survival and graft survival rates of renal transplant recipients on belatacept were similar to those of cyclosporine-treated recipients over 3 years, but with statistically better renal function and cardiovascular/metabolic disease risk status (40–44). Schwarz et al. (45) conducted a trial of belatacept for liver transplantation in 15 patients, which was terminated due to graft dysfunction with acute rejection at approximately 10 weeks. Interestingly, in another study, belatacept was reportedly safe and effective in hepatitis C-positive patients with renal insufficiency and for use as a bridge to renal rehabilitation (46). In rat liver transplantation models, CTLA4 signaling is essential for inducing immune tolerance (23). However, in a phase II clinical trial of adult liver transplantation, belatacept treatment resulted in a higher incidence of acute rejection and graft loss (24). Perhaps the “benefits” of belatacept in liver transplantation will be shown in appropriate patient selection and trial design.

### ICOS/ICOSLG Costimulatory Pathway

ICOS is an inducible T cell costimulatory molecule of the Ig superfamily with strong structural similarity to CD28 and CTLA4 (47). It is expressed on activated T cells and its expression persists in effector and memory T cells. The B7 family member ICOSLG is structurally related to B7-1/B7-2. It is expressed on B cells, macrophages and dendritic cells; its expression can also be induced on non-lymphoid cells, including endothelial and pulmonary epithelial cells (48). ICOS binds only with ICOSLG, but not B7-1 or B7-2 (49, 50). The ICOS/ICOSLG pathway is critical for T cell-dependent B cell responses (51, 52). ICOS costimulation can enhances T-cells activation, proliferation, differentiation and effector functions. Treatment with anti-ICOS antibodies can prolong the survival of cardiac allografts (53). The timing of ICOS blockade is a key factor; only delayed blockade can inhibit the production of CD8+ T cells and statistically prolong the survival time of allografts (54). Treatment with anti-ICOS antibodies in combination with anti-CD154 antibodies or CTLA4Ig can prolong the survival of heart allografts and prevent chronic rejection (55). Some studies have shown that the survival of rat liver allografts can be prolonged by injecting anti-ICOS antibody after surgery (25). When combined with FK506, an anti-ICOS antibody synergistically prevents rejection after liver transplantation and induces graft tolerance (26). In addition, activation of the ICOS pathway can be inhibited by RNA interference, which prevents acute rejection and prolongs the survival of grafts by promoting T cell apoptosis and suppressing the production of cytokines by T lymphocytes (27). Considering that ICOS appears to work independently of CD28, blocking the ICOS/ICOSLG pathway in combination with the CD28/B7 pathway may be as a potential therapeutic strategy, but the ICOS/ICOSLG blocking drugs or clinical trial have not yet been studied in human liver transplantation (56).

## TNF-Related Superfamily Costimulatory Pathways

### CD40/CD154 Costimulatory Pathway

CD40 is a member of the TNF receptor family, which is expressed in APCs, including B cells, macrophages, and dendritic cells (DCs), as well as in endothelial cells, fibroblasts, and smooth muscle cells (57). CD40 mainly binds to CD154, which is expressed on activated T cells. CD154 also belongs to the TNF superfamily; both CD40 and CD154 are type II transmembrane proteins. In addition to playing an important role in B cell activation and Ig class conversion, the CD40/CD154 costimulatory pathway is important for costimulating T cell immune responses (58). CD40/CD154 interactions are also critical in T cell-dependent humoral immune responses and T cell-mediated activation of DCs and macrophages (59). The interaction between CD40 on T cells and CD154 on APCs lead to the maturation of DCs, which increases the production of cytokines and costimulatory molecules and enhances their ability to promote T effector cell differentiation (60). This pathway affects the function of many immune cells that are critical to the adaptive immune response, and studies in animal transplant models have shown considerable promise. Targeting CD154 prevents acute rejection and induces tolerance in some transplant models (61). In a model of mouse skin and heart transplantation, treatment with anti-CD154 prolongs graft survival (62, 63). In an NHP model, blocking CD154 leads to long-term survival of renal allografts and the loss of donor-specific mixed lymphocyte reactivity (64). When used in combination with CTLA4Ig, CD40/CD154 blockade had synergistic effects, on the enhancement of long-term skin and heart graft survival (65, 66). However, thromboembolic complications related to the anti-CD154 antibody were later reported in NHP research (67). It is now believed that the binding of the Fc domain of the anti-CD154 antibody to the Fc receptor of platelets contributes to platelet aggregation (68). Therefore, the current approaches to targeting this pathway mainly focus on the use of CD40-blocking antibodies.

Treatment with an anti-CD40 monoclonal antibody is an effective alternative method to block the costimulatory CD40/CD154 signal without interfering with platelet aggregation. ASKP1240 is a fully humanized inhibitory monoclonal antibody against CD40, which can block the CD40/CD154 interaction and inhibit cell-mediated and humoral immune responses without immunogenic and thromboembolic complications (69). A trial in NHPs showed that monotherapy with ASKP1240 increases the survival rate of liver grafts without the occurrence of thromboembolism, and monkeys showed good tolerance (28). In a 2017 study of a liver xenotransplantation model, the use of a blocking anti-CD40 monoclonal antibody prolonged the survival of xenografts (29). Other CD40 antibodies, such as 4D11, HCD122, and 2C10R4, have been effective in heart and kidney transplantation studies, but they have not been tested in liver transplantation studies.

### OX40/OX40L Costimulatory Pathway

The expression of the TNF superfamily member OX40 on activated T cells is time-dependent (70). OX40 is essential for the regulation of T cell proliferation, differentiation, survival, and cytokine production (71). The expression of its ligand OX40L is induced on activated T cells and APCs, such as DCs, macrophages, and B cells, but also some endothelia and mast T cells. OX40-OX40L costimulatory pathway has been shown to be involved in the regulation of Th cells differentiation. Although CD28 signaling up-regulates the expression of OX40 on T cells, OX40 costimulation does not depend on a complete CD28 signal (72). Blocking the OX40/OX40L pathway alone had little effect in an allograft model (73). However, OX40/OX40L pathway blockers prolonged allograft survival time in CD28/CD40 dual-gene knockouts or in transplantation models featuring CD28/B7-1 blockers (74, 75). However, OX40/OX40L costimulatory blockade inhibited skin allograft rejection not by inhibiting T cell activation and proliferation, but by preventing the trafficking of peripheral lymph node effector T cells into the grafts (76). Combination therapy using OX40L blockers with traditional costimulatory blockers effectively prevents the allo-reactive T cell responses that impede long-term graft function and survival (47). Blocking the OX40/OX40L pathway with OX40Ig inhibits the rejection of liver allografts and induces immune tolerance in rats by reducing IL-2 expression (30). However, there have been no any clinical trials of OX40/OX40L pathway blockade in transplantation.

### TNF Receptor Superfamily Member 9/TNF Superfamily Member 9 Costimulatory Pathway

TNF receptor superfamily member 9 (also known as 4-1BB or CD137) is a transmembrane protein expressed on T cells, DCs, and B cells. It reaches peak expression after T cell activation. Its ligand TNF superfamily member 9 (also known as 4-1BBL or CD137L) is expressed on APCs, including mature DCs, macrophages, and activated B cells, but not on resting or activated T cells (77). The 4-1BB/4-1BBL costimulatory signal can activate T cells independently of the CD28 signal (78), and 4-1BB can provide sufficient costimulation to drive T cell activation. The role of the 4-1BB/4-1BBL costimulatory pathway in transplantation varies depending on the model, as uncovered using antagonistic or agonistic anti-4-1BB monoclonal antibodies or gene silencing of 4-1BB. In a mouse model of graft-versus-host disease, treatment with an agonistic anti-4-1BB monoclonal antibody exacerbated cytotoxic CD8^+^ T cell-mediated tissue damage and accelerated the rate of rejection of heart allografts or skin grafts (79). However, blocking the interaction of 4-1BB/4-1BBL with an antagonistic 4-1BB monoclonal antibody prolonged allograft survival time and helped prevent allograft rejection (31). It has been reported that silencing 4-1BB with RNA interference or blocking the pathway with an anti-4-1BBL monoclonal antibody can inhibit or limit acute rejection in rat liver transplantation (32, 33).

### TNF Receptor Superfamily Member 18/TNF Superfamily Member 18 Costimulatory Pathway

TNF receptor superfamily member 18 (also known as glucocorticoid induced tumor necrosis factor related receptor or GITR) is a type I transmembrane protein that can be expressed on T lymphocytes, NK cells, and APCs. Regulatory T cells highly express GITR, which can also be expressed at low levels on resting T cells; however, the expression of GITR is up-regulated when T cells are activated, especially in the presence of the CD28 signal (80). Its ligand TNF receptor superfamily member 18 (also known as GITRL) is mainly expressed on APCs after stimulation through Toll-like receptors. GITR activation is a positive costimulatory signal for CD4^+^ and CD8^+^ T cells, leading to enhanced proliferation, survival, and cytokine production (81). In addition, GITR-induced signaling is important for regulatory T cell-mediated inhibition of effector T cell activity and the prevention of autoimmune diseases. Shimizu J et al. (82) found that increased expression of GITR in T cells impairs allograft tolerance and self-tolerance. Wei et al. (83) showed that GITR expressed on Kupffer cells may mediate acute rejection of rat liver grafts. However, the role of the GITR/GITRL pathway in transplantation requires further investigation.

## Other Pathways

### TIM Family Molecules

The TIM family of genes encodes type 1 glycoproteins that share a common Ig V-like domain, mucin-like domain, single transmembrane domain, and cytoplasmic domain (84). The TIM gene family consists of 8 members in mice; the 3 human TIM genes are most similar to mouse TIM-1, TIM-3, and TIM-4. As a novel family of costimulatory molecules, the TIM gene family plays an important role in the activation and differentiation of Th cells (85). TIM-1 (also known as HAVCR1 or KIM1) is not expressed on naive CD4^+^ T cells, but it is expressed after TCR stimulation, preferentially on Th2 cells (34). TIM-1 is not only necessary for regulating Th1 and Th2 immune responses, it also regulates Th17 and regulatory T cells. Agonism of TIM-1 with the high-affinity monoclonal antibody 3B3 promoted the expansion of antigen-specific T cells expressing Th1 and Th17 cytokines and blocked allograft tolerance (34, 35). However, the use of the blocking monoclonal antibody MT1-10, which has a lower affinity for TIM-1, prolonged the survival of completely mismatched cardiac allografts and induced tolerance in combination with rapamycin (86).

Although it was originally identified in Th1-differentiated cells, TIM-3 has a wide range of expression and is the first among the TIM family of proteins that was discovered. In addition to its expression on Th1 and Th17 cells, it is constitutively expressed on DCs, macrophages, NK cells and mast cells (84). Like other TIM family members, TIM-3 is a phosphatidylserine receptor; it can bind multiple ligands, including galectin-9, phosphatidylserine, high mobility group box 1, and CEA cell adhesion molecule 1 (84, 87–89). As a negative costimulatory molecule, TIM-3 dampens Th1 and Th17 responses after binding galectin-9, thereby playing an important role in immune and inflammatory responses. It can promote apoptosis and inhibit the immune response mediated by Th1 cells. In a cardiac allograft transplantation model, blocking TIM-3/galectin-9 costimulatory signal transduction with an anti-TIM-3 monoclonal antibody (RMT3-23) accelerated rejection (90), in a process characterized by the promotion of Th1/Th17 polarization, inhibition of regulatory T cell differentiation, and promotion of donor-specific alloantibody production. In contrast, the application of exogenous galectin-9 prolonged the survival of skin and heart allografts (91, 92), and combination therapy with rapamycin promoted allograft tolerance (93). So far, human transplantation studies have focused on the use of Tim-3 as a marker of Th1 activation and rejection.

TIM-4 is mainly expressed on APCs, including CD11c^+^ DCs and macrophages, but not on T cells (94). TIM-4 was originally thought to be a ligand of TIM-1 that promoted T cell proliferation; however, it was later demonstrated that direct interaction between TIM-1 and TIM-4 was achieved by bridging exosomes (95). The specific effect of TIM-4 on T cell activation remains unclear, and *in vitro* studies using the TIM-4Ig fusion protein have shown conflicting results. The use of the TIM-4Ig fusion protein can enhance TIM-4 signal transduction and increase the proliferation of activated T cells, but has the opposite effect on naive T cells (96–98). Blocking TIM-4 ameliorated acute rejection injury after liver transplantation in rats and down-regulated the expression of TNF‐α, IFN‐γ, CCL2, and CXCL2 in allografts. When combined with exogenous TGF-β, it further ameliorated acute rejection injury and increased graft survival time (36).

### Integrin Subunit Alpha L/Intracellular Adhesion Molecule 1 Costimulatory Pathway

Integrin subunit alpha L (also known as lymphocyte function-associated antigen 1 or LFA-1) is an adhesion molecule found on the surface of T cells, which belongs to the integrin family of cell adhesion factors. When it binds intercellular adhesion molecule 1 (ICAM1) expressed on endothelial cells, LFA-1 can provide the costimulatory second signal and promote the activation and proliferation of T cells (99). Some studies have shown that blocking the interaction between LFA-1 and ICAM1 with anti LFA-1 and anti-ICAM1 monoclonal antibodies prolonged the survival time of mouse skin, heart, and islet allografts (100–102). Earlier studies showed that the use of anti-ICAM1 and anti-LFA-1 antibodies prolonged the survival of rat liver allografts, but did not induce permanent tolerance (103). When combined with donor-specific blood transfusion, LFA-1/ICAM1 blockade induced tolerance in 80% of rats (37). Currently, few clinical trials have investigated blocking this costimulatory pathway in liver transplantation, and its future role in liver transplantation remains unclear.

## Conclusion

Traditionally, the induction of allograft tolerance has been regarded as the “holy grail” of transplantation immunology as graft can survive a long time in patients with tolerance. However, for most liver transplant recipients, it is still very difficult to withdraw immunosuppressants and achieve immune tolerance. T cell-mediated rejection after liver transplantation is a complex and dynamic process. The relative strength of the costimulatory and coinhibitory signals activated after transplant determines how T cells respond to allografts. As the key second signal, costimulatory pathways are essential in the activation of T cells, especially CD28/B7 costimulatory signal pathway. Although belatacept has achieved considerable results in clinical renal transplantation since it was approved by FDA, its clinical trials results in liver transplantation are not very satisfactory. Considering the complex mechanisms involved in the immune response to liver allograft transplantation, blocking a single costimulatory pathway may not be sufficient to induce tolerance. Besides, further clinical trials may be needed to compare different costimulatory blockers to understand their respective advantages, and we anticipate that blocking multiple costimulatory pathways in combination with coinhibitory signaling pathways may be the optimal regimen to achieve the true transplant tolerance in humans.

## Author Contributions

All authors listed have made a substantial, direct and intellectual contribution to the work, and approved it for publication.

## Funding

This work was supported by the National Natural Science Foundation of China (81671958 and U1604282) & the Tackling Plan for Scientific and Technological in Medicine of Henan Province (SBGJ2018002) and the Supporting Plan for Scientific and Technological Innovative Talents in Universities of Henan Province (19HASTIT003).

## Conflict of Interest

The authors declare that the research was conducted in the absence of any commercial or financial relationships that could be construed as a potential conflict of interest.
